# Parietal epithelial cell dysfunction in crescentic glomerulonephritis

**DOI:** 10.1007/s00441-021-03513-9

**Published:** 2021-08-28

**Authors:** Milagros N. Wong, Pierre-Louis Tharaux, Florian Grahammer, Victor G. Puelles

**Affiliations:** 1 III. Department of Medicine, University Medical Center Hamburg-Eppendorf, Hamburg, Germany; 2 Institut National de la Santé et de la Recherche Médicale (Inserm), Paris Cardiovascular Center – PARCC, Université de Paris, Paris, France

**Keywords:** Crescentic glomerulonephritis, Immune system, Parietal epithelial cells, Podocyte gain, Parietal cell activation

## Abstract

Crescentic glomerulonephritis represents a group of kidney diseases characterized by rapid loss of kidney function and the formation of glomerular crescents. While the role of the immune system has been extensively studied in relation to the development of crescents, recent findings show that parietal epithelial cells play a key role in the pathophysiology of crescent formation, even in the absence of immune modulation. This review highlights our current understanding of parietal epithelial cell biology and the reported physiological and pathological roles that these cells play in glomerular lesion formation, especially in the context of crescentic glomerulonephritis.

## Introduction

Chronic kidney disease (CKD) has been recognized as a global health problem of pandemic proportions (GBD Chronic Kidney Disease Collaboration 2020). CKD eventually progresses to end-stage kidney disease (ESKD), reaching a clinical stage that requires renal replacement therapy (i.e. dialysis) or kidney transplantation in order to prolong the patient’s life. While over 10 million people worldwide require dialysis or transplantation, many do not receive these interventions due to financial constraints or lack of resources (Himmelfarb et al. 2020). Furthermore, given that dialysis does not provide a cure and there is a great disparity between the number of patients requiring transplants and the number of available organs (Hippen et al. 2009), there is an urgent need for the development of additional therapeutic strategies that may prevent or slow-down CKD/ESKD.

Crescentic glomerulonephritis (cGN) is one of the most aggressive conditions that can quickly lead to CKD/ESKD (Jennette and Thomas 2001). cGN is characterized by the presence of extensive and destructive glomerular cellular crescents, usually in more than 50% of glomeruli, which explains the sudden and progressive loss of renal function. The pathological definition of crescents varies depending on the specific disease, but cellular crescents are commonly defined as two or more layers of proliferating cells in Bowman’s space. Previous evidence suggests that parietal epithelial cells (PECs) are the main cell type populating crescents (Smeets et al. 2009a) as they undergo an activation process characterized by increased capacity for proliferation, migration and production of extracellular matrix (Ohse et al. 2009a).

PEC activation in cGN usually occurs during a complex immune response, namely, macrophage and T cell infiltration. Multiple studies have shown that modulating the immune system can both exacerbate and inhibit crescent formation (Krebs et al. 2017) suggesting potential interactions between PECs and the immune system. Given the role of PECs as effector cells in crescent formation, it is likely that immune reactions may serve as a trigger leading to PEC activation. Furthermore, crescents are also characterized by immune cell infiltration, which is dependent on the integrity of Bowman’s capsule (BC) (Chen et al. 2018), a structure that is in immediate contact with PECs and that may be directly affected in this activation process.

While the involvement of PECs in crescent formation is well established, basic physiological roles of PECs remain incompletely understood. It has been proposed that PECs may serve as a barrier to prevent ultrafiltrate leakage into the interstitium (Ohse et al. 2009a) and to prevent immune cell infiltration into the glomerulus (Chen et al. 2018). Furthermore, PECs have primary cilia (Arakawa and Tokunaga 1977). Given that PECs are continuously exposed to flow from the glomerular filtrate, it has also been proposed that these cilia may serve as chemical and mechanical sensors (Ohse et al. 2009b), which could facilitate inter-cellular communication without direct contact. Additionally, PECs have been proposed as cellular reservoirs of podocytes that can contribute to postnatal podocyte gain (Shankland et al. 2017), a topic that still remains under continuous debate (Moeller and Tharaux 2019). However, recent studies have shown evidence of podocyte loss in humans (Zimmermann et al. 2021) and mice (Henique et al. 2017; Puelles et al. 2019a) during cGN, suggesting that, in this condition, the potential for postnatal podocyte gain is limited and does not seem to be able to compensate for podocyte loss.

In summary, this review will highlight current evidence regarding the central role of PEC activation and functional impairment in the origin and progression of cellular crescents and thereby cGN.

### Immune triggers of crescent formation

Most forms of cGN are pathophysiologically regarded as immune-mediated (Couser 2012; Anders and Fogo 2014). However, in most of the cases, the specific etiology remains unknown. It has been hypothesized that crescent formation may be the result of triggers from both the adaptive and the innate immune system, leading to diverse clinical and pathologic manifestations (van den Berg and Weening 2004; Kitching and Hutton 2016). For more comprehensive reviews on the role of immune cells in cGN, please refer to Krebs et al. (2017), Tang et al. (2019), Antonelou et al. (2020) and Kurts et al. (2020).

Briefly, human cGN is characterized by glomerular accumulation of neutrophils, monocytes, T cells and macrophages (Hooke et al. 1987). Based on this observation, multiple studies have suggested that these immune cells play key roles in the initiation of immune responses leading to the formation of cellular crescents (Neale et al. 1988).

Neutrophil infiltration is observed in the biopsies of patients with cGN irrespectively of the cause (Suh et al. 1999). Neutrophil recruitment within glomerular capillaries following IgG deposition has been shown to be further enhanced by transgenic expression of the human Fc receptor Fc gamma RIIA, which promotes glomerular neutrophil accumulation (Nishi et al. 2017). Through MPO-mediated oxidative activity, release of proteases, activation of the complement cascade and release of NETs that recruit red blood cells and promote fibrin deposition, the increased dwell time of neutrophils in glomerular capillaries promotes endothelial injury. Multiphoton and spinning disk confocal intravital microscopy have revealed that the major effect of acute inflammation is to increase the duration of leukocyte retention in the glomerulus. Furthermore, multicellular intravascular patrolling involving both monocytes and neutrophils was uncovered (Devi et al. 2013). Monocytes patrol both uninflamed and inflamed glomeruli using beta2 and alpha4 integrins and CX3CR1. Monocyte depletion reduced glomerular injury, demonstrating that these cells promote inappropriate inflammation in this setting. Monocyte depletion also resulted in reductions in neutrophil recruitment and dwell time in glomerular capillaries and in reactive oxygen species generation by neutrophils, suggesting a role for cross-talk between monocytes and neutrophils in induction of cGN (Finsterbusch et al. 2016).

CD4 + T cells play a key effector role due to their ability to recruit macrophages. Interestingly, CD4 + T cell depletion in a rodent model of cGN effectively prevented glomerular macrophage recruitment and crescent formation (Huang et al. 1994). Furthermore, Heymann et al. (2009) showed the ability of CD4 + T cells to orchestrate the formation of focal periglomerular mononuclear infiltrates, which play a key role in the invasion of CD8 + T cells through BC, amplifying crescentic lesion formation (Chen et al. 2018).

Previous studies have shown that T helper type 1 (Th1) cytokine deficiencies (e.g. IL-12 (Kitching et al. 2005) and IFN-γ (Kitching et al. 1999a)) as well as blocking Th1 cytokines (Tipping and Holdsworth 2006) attenuate the development of crescents. In addition, administration of IL-12 exacerbates experimental cGN, which confirms the key role of this cytokine (Kitching et al. 1999b). Importantly, mice lacking RORγt are unable to produce T helper 17 (TH17)-mediated immune responses, which protects mice against cGN (Krebs et al. 2017). Interestingly, deficiencies in the p19 subunit of IL-23 and IL17A lead to attenuation of experimental cGN (Paust et al. 2009). Together, these studies represent excellent examples of a direct effect of T cells in the pathogenesis of cellular crescent formation.

Yet, the role of immune cells in cGN is not black and white. For instance, mice lacking the p40 subunit of IL‑23 and IL‑12, the p19 subunit of IL‑23 or the p35 subunit of IL‑12 were only protected in the absence of IL‑23 signalling, while the presence or absence of IL‑12 had no influence on disease onset (Ooi et al. 2009). Another example can be found in the process of dendritic cell maturation during experimental cGN, which is generally mediated by the transcription factor nuclear factor-κB (NF-κB). In murine cGN, pharmacological inhibition of NF-κB diminished the maturation of DCs, but the subsequent loss of regulatory T cells exacerbated multiple features of crescentic disease (Gotot et al. 2016).

**Fig. 1:**
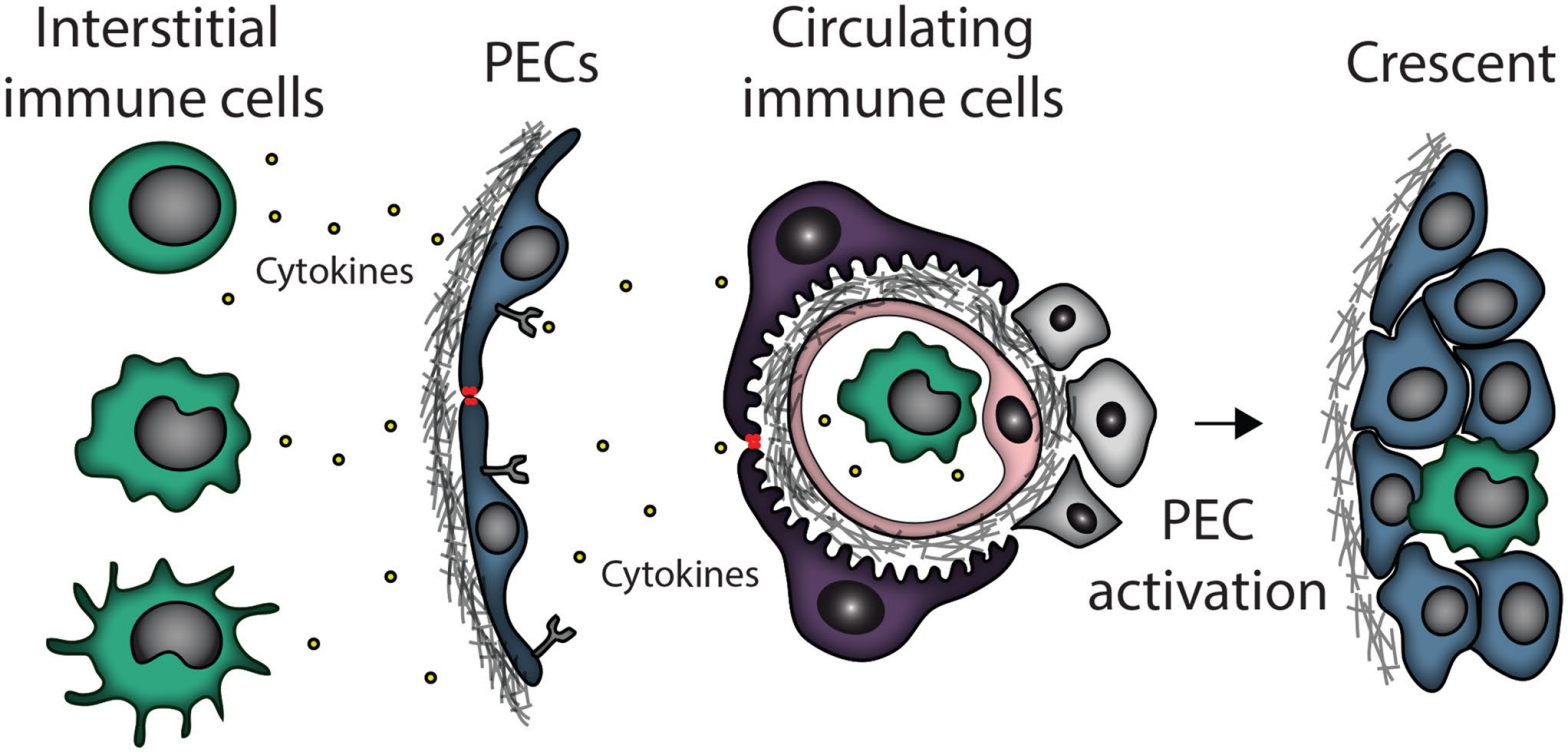
Immune responses trigger parietal epithelial cell (PEC) activation. Both interstitial and circulating immune cells are able to produce mediators of PEC activation, which could lead to crescent formation

These examples highlight that complex immune-mediated processes can serve as powerful triggers for crescent formation and evolution (Fig. 1). However, their direct effects on PEC activation remain unclear.

### PEC dysfunction

**Fig. 2:**
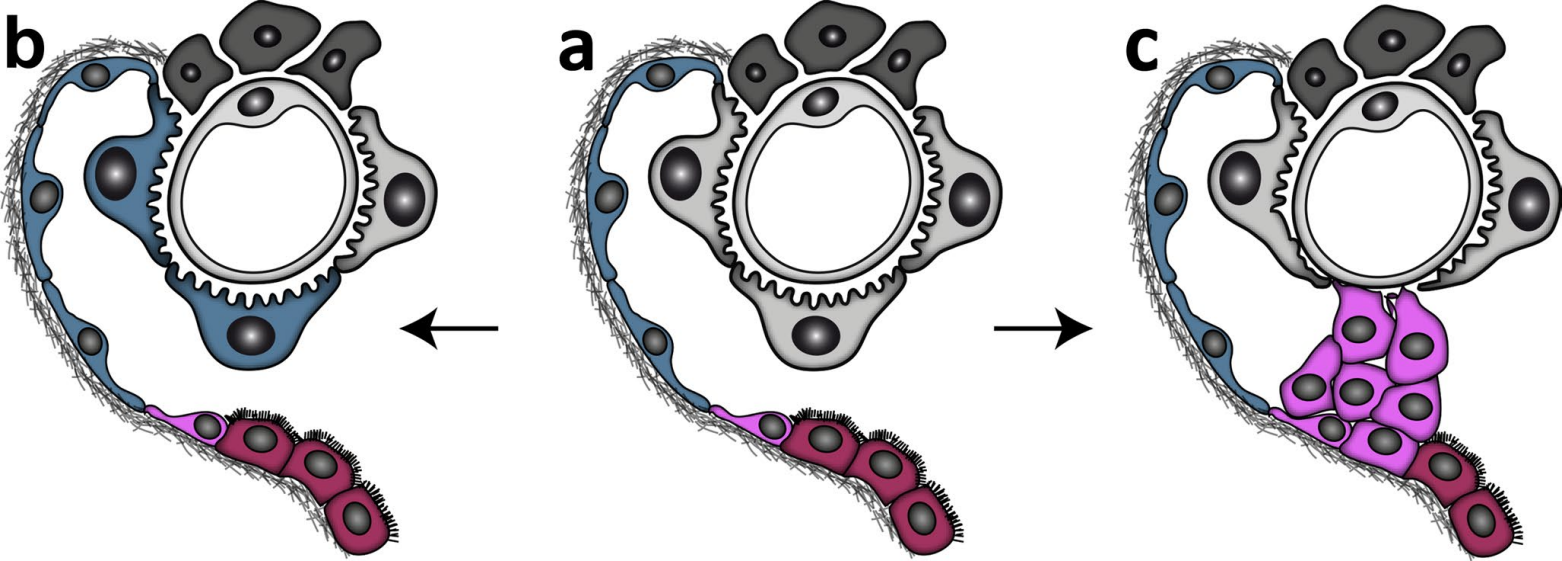
Functional and pathological roles of PECs. **a** Glomerular schematic showing the 3 different subtypes of PECs: flat (blue), intermediate (pink) and cuboidal (red). **b** PECs potentially contribute to postnatal podocyte gain either as active progenitors or serving as a functional reservoir. **c** Activated PECs form cellular crescents via an activation process characterized mainly by an increased capacity to migrate and proliferate

Epithelial cells lining on Bowman’s capsule (BC) are referred to as PECs. Although this glomerular cell type was first described in the 1800s (Bowman 1842), only recently, PECs gained attention due to their potential contribution to postnatal podocyte gain and proven role in glomerular lesion formation (Fig. 2).

During nephrogenesis, PECs and podocytes develop from the metanephric mesenchyme that is induced by the ureteric bud. Both cell types undergo a mesenchymal to epithelial transition forming the renal vesicle, which after a series of elongations and invaginations, generates S-shaped bodies. In the transition between S-shaped body and capillary loop stage, PECs differentiate into podocytes through the upregulation of podocyte-specific genes and the de novo expression of the cyclin-dependent kinase inhibitor p27, and downregulation of PAX2 (Shankland et al. 2014).

### PECs and podocyte gain

Podocytes are post-mitotic highly specialized epithelial cells unable to complete cytokinesis (Kriz et al. 1995; Lasagni et al. 2013) with a limited regeneration potential (Puelles and Moeller 2019b). It has been shown that podocyte loss is sufficient for the initiation of glomerulosclerosis (Kim et al. 2001; Wharram et al. 2005; Puelles et al. 2019a) and has been proposed as a unifying principle of glomerular disease (Wiggins 2007). While podocyte loss may be the main trigger for glomerulosclerosis, PECs serve as effector cells that initiate the formation of segmental lesions (Dijkman et al. 2005; Lazareth et al. 2020; Kuppe et al. 2019). However, is it possible that PECs can also play a role in some form of postnatal podocyte gain?

Sagrinati et al. characterized the expression of CD24 and CD133 in PECs, which initiated the hypothesis that PECs could exhibit stem cell-like properties (Sagrinati et al. 2006). Subsequent work by Ronconi et al. (2009) suggested that these cells may act as podocyte progenitors. Furthermore, Appel et al. (2009) showed using genetic lineage tracing that, in juvenile mice, a small number of PECs migrated into the glomerular tuft and co-expressed podocyte markers (e.g. nephrin and WT-1). Both of these studies sparked up an interesting debate regarding the possibility of podocyte regeneration, something that until then was considered impossible. Three main theories remain: (1) PECs are a limited, but available source of podocyte progenitors in the adult period; (2) PECs represent a limited reservoir of differentiated podocytes that migrate to the tuft when sufficient space is available (i.e. during glomerular growth); or (3) PECs can acquire podocyte markers but do not functionally replace podocytes.

Over time, new arguments were introduced, for example, the co-expression of podocyte and PEC markers in glomerular cells (Ohse et al. 2010), the expression of podocyte markers in PECs (i.e. during aging (Puelles et al. 2016) and diabetic nephropathy (Andeen et al. 2015), and podocyte/PEC phenotype control via miR-193a (Kietzmann et al. 2015). Over the years, some studies failed to identify PECs as meaningful contributors to the podocyte pool during adult life (Wanner et al. 2014; Berger et al. 2014), and others have confirmed and expanded the initial findings (Eng et al. 2015; Romoli et al. 2018; Kaverina et al. 2019). For more extensive discussions on this topic, we refer to Moeller and Tharaux 2019; Puelles and Moeller 2019; Shankland et al. 2017; Mazzinghi et al. 2016.

### Classical definition of PEC activation

In parallel to the first studies suggesting that PECs could be a potential source of new podocytes, Smeets et al. (2009b) proposed that PECs (at the time referred to as “renal progenitors”) were involved in the development of glomerular lesions, which included cellular crescents. This observation was also made in the mid-eighties by Guettier et al. (1986) as PECs were clearly identified as the main components of these lesions. Years later, lineage tracing experiments in rodents confirmed that PECs are the main cell type involved in the origins of two key patterns of glomerular pathology: segmental glomerulosclerosis and crescents (Moeller and Smeets 2014).

It has been proposed that PECs undergo a process of activation with a classical cascade, including increased potential for proliferation, migration, production of extracellular matrix and de novo expression of certain markers (i.e. CD44 and CD9) (Lazareth et al. 2020). While crescent formation involves an initial stage of pronounced migration and proliferation, followed by a pro-fibrotic phase (Smeets et al. 2009a), segmental glomerulosclerosis tends to feature limited migration and proliferation but features marked extracellular matrix deposition (Smeets et al. 2011). In our opinion, this difference alone could suggest that PEC activation may be regulated by different signals that may shift the process from proliferative to fibrotic.

### Not all PECs are the same

Interestingly, in normal glomeruli, “parietal podocytes” are described at the intersection of PEC and podocytes as cells expressing, both markers of PEC and markers of podocytes (Appel et al. 2009; Bariety et al. 2006; Gibson et al. 1992; Ronconi et al. 2009). In rats, such “transitional” PECs were found to express NCAM, Claudin1 and WT1 (Benigni et al. 2011). The significance of such findings is unclear, but observations report an increased number of these parietal podocytes during rodent models of glomerular diseases with podocyte loss (Benigni et al. 2011; Ohse et al. 2010; Pichaiwong et al. 2013). Based on morphology, Kuppe et al. (2019) recently showed that there are different subtypes of PECs, namely, flat, intermediate and cuboidal, which have different activation potential. For example, while intermediate and cuboidal PECs seem to be particularly sensitive to activation in models of segmental glomerulosclerosis, flat PECs appear to be more stable. It remains unclear if these morphological differences and different activation potential also reflect different signalling pathways that are selectively activated in each PEC subtype.

One of the main features of PECs during crescent formation is their capacity to proliferate. A low level of proliferative activity has been reported in PECs under baseline conditions (Pabst and Sterzel 1983). Flat PECs express Src-suppressed protein kinase C substrate (SSeCKS), a multivalent scaffolding A kinase anchoring protein (Schulte et al. 2014) that is able to regulate cyclin D1 activity, which has been linked to an increased proliferative activity in intermediate PECs during the initiation of segmental glomerulosclerosis (Kuppe et al. 2019). Importantly, Burnworth et al. (2012) provided evidence that SSeCKS knockout mice showed PEC hyperplasia without any other stress stimuli and developed more severe cGN, characterized by increased PEC proliferation, which can be attributed to nuclear translocation of cyclin D1 upon activation. Together, these findings suggest that our definition of PEC activation may not only need to consider differential triggers but also different activation profiles and different response states per PEC subtype.

### Molecular basis for PEC activation

De novo expression of the cell surface glycoprotein CD44 has been used as a central feature of PEC activation (Smeets et al. 2009a, 2011; Okamoto et al. 2013; Kim et al. 2016). This concept has also been extended to clinical scenarios, where expression of CD44 by PECs has even been used to differentiate between minimal change disease and focal segmental glomerulosclerosis (Smeets et al. 2014) and as a marker of renal function deterioration in paediatric patients (Froes et al. 2017).

A recent report characterized the role of tetraspanin CD9 in the development of glomerulosclerosis and crescents (Lazareth et al. 2019). Using PEC-specific genetic deletion of CD9, Lazareth et al. showed that selective PEC inactivation was sufficient to abolish lesion formation, even in the presence of significant podocyte loss. Interestingly, Cd9 gene targeting abrogated expression of CD44 in PECs both in crescentic GN and FSGS models, suggesting that de novo expression of CD9, is a requirement for further CD44 expression and formation of extracapillary lesions (Lazareth et al. 2019). Furthermore, the authors also showcased the capacity of PECs to sense local changes in chemoattractants (i.e. PDGF-β and HB-EGF), linking PEC activation to factors emanating from the injured tuft.

Djudjaj et al. (2016) showed that local upregulation of macrophage migration inhibitory factor (MIF) and its receptor complex CD74/CD44 mediated PEC activation and thereby crescent formation in cGN. In subsequent studies using CD44 global knockout mice, Roeder et al. (2017) and Eymael et al. (2018) demonstrated a significant attenuation of glomerulosclerosis and crescent formation, confirming the key role of CD44 in PEC activation.

In an intriguing study, Kuppe et al. (2017) characterized the action of glucocorticoids on activated PECs in cGN. While glucocorticosteroid administration attenuated cGN as expected, glucocorticosteroid receptor deficiency and pharmacological glucocorticosteroid antagonism also ameliorated crescent formation in mice. This duality may provide some experimental explanations for therapy resistance and relapses in cGN, which await future clinical validation.

### PEC activation without immune triggers

To date, there is no doubt that immune triggers play an important role in crescent formation. However, evidence shows that PEC activation in the absence of these triggers might be possible as well.

A role for endothelial damage and activated coagulation cascade involving the thrombin receptor PAR-1 was shown in experimental cGN (Cunningham et al. 2000), suggesting a potential mechanistic link between glomerular fibrinoid necrosis and PEC recruitment. Similarly, Morigi et al. (2016) showed in a mouse model of protein overload that PEC activation occurred in response to podocyte depletion, which triggered complement activation, and glomerulosclerosis. This was mirrored in human renal biopsies, showing concomitant PEC activation and glomerular C3/C3a deposition, suggesting a potential role of C3/C3a in the development of PEC activation.

It has been reported that mice or rats that constitutively lack T cells are still capable of developing cGN (Kusuyama et al. 1981; Sato et al. 1991), and crescent formation can be modulated by intrinsic glomerular cells (i.e. podocytes) through the common gamma chain, interleukin-2 receptor β subunit, and IL-15, independent of immune responses (Luque et al. 2017).

Interestingly, Ryu et al. (2012) showed that glomerular vascular injury and GBM breaks in experimental, and human Alport nephropathy causes plasma leakages that can trigger crescent formation. In addition, Chang et al. (2012) reported that increased albumin uptake by PECs can lead to apoptosis through changes in extracellular signal-regulated kinase 1 and 2.

Importantly, Sicking et al. (2012) performed an elegant study using a mouse model that expressed a diphtheria toxin receptor in PECs. Administration of diphtheria toxin led to selective PEC ablation and overt crescent formation, in the absence of an identifiable immune trigger.

Together, these studies suggest that immune responses are not a requirement for crescent formation, which reinforces the key role of PEC activation in cGN.

### Impaired PEC function

Taugner et al. (1976) showed using electron microscopy that PECs form intercellular tight junctions, which typically form impermeable barriers between adjacent cells, preventing the passage of molecules. Interestingly, Ohse et al. (2009b) provided evidence that these tight junctions were no longer visible during the course of cGN, which correlated with functional studies showing that PECs together with their corresponding basement membrane serve as a second barrier to protein that is dysregulated upon activation.

In addition, PECs sit on a multi-layered basement membrane, which is thickened during PEC activation (Smeets et al. 2011; Holderied et al. 2015). Interestingly, a landmark study by Chen et al. (2018) characterized the Bowman’s capsule (BC) as a protective niche for podocytes from cytotoxic CD8 + T cells. Thus, it is likely that the integrity of BC could determine immune cell infiltration to the crescents and subsequent podocyte injury and depletion. Importantly, podocyte loss in experimental cGN has been identified using lineage tracing and optical clearing (Puelles et al. 2019c) as well as in human biopsies of ANCA-GN patients (as an example of cGN) using deep learning (Zimmermann et al. 2021), which could be explained by basement membrane ruptures leading to direct contact between PECs, podocytes and immune cells, and perhaps a failure of PECs to successfully replenish lost podocytes during cGN.

**Fig. 3:**
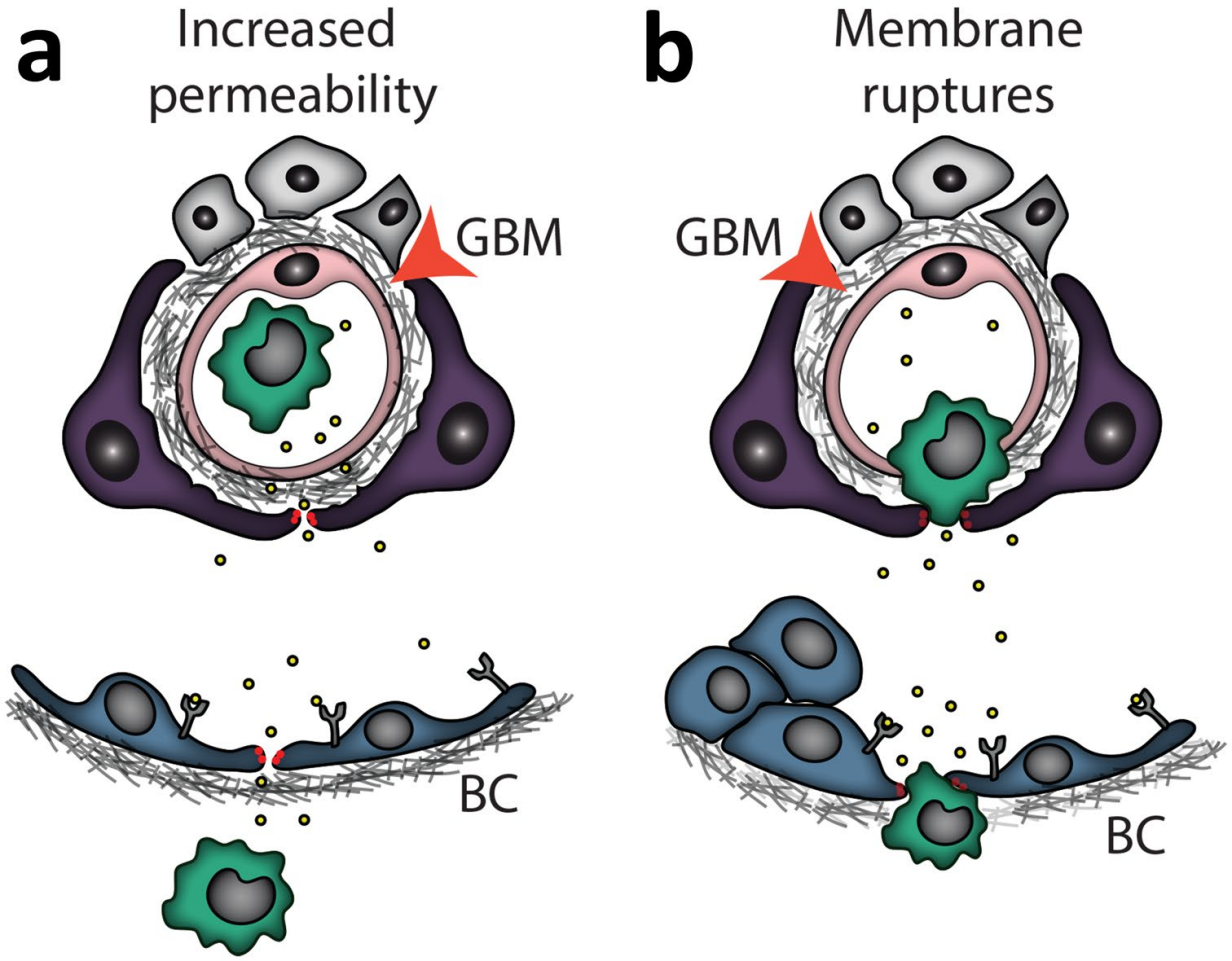
Membrane integrity and PEC activation. PEC activation may also play a role in basement membrane integrity, as increased permeability facilitates the passage of circulating or interstitial signals that can reach PECs (**a**). Membrane ruptures will not only facilitate signals but also translocation of immune cells to reach out and have direct interactions with PECs and other glomerular cells (i.e. podocytes) (**b**). GBM, glomerular basement membrane; BC, Bowman’s capsule

Together, these findings summarize how membrane integrity can directly affect PEC function and contribute to facilitate triggers of PEC activation and additional features of cGN (i.e. immune infiltration in crescentic lesions and podocyte loss) (Fig. 3).

#### Conclusion

The evidence presented in this review suggests that we should consider a broader definition for PEC activation, that not only considers classical activation steps such as increased proliferation, migration and production of extracellular matrix, but also integrates novel signalling pathways directly involved in PEC activation and active dysregulation of physiological roles (i.e. second barrier, protective niche and potential podocyte reserve). As we unravel new features of PEC activation, especially those related to impaired function, the use of the term PEC dysfunction will become more appropriate to describe this set of complex biological processes.

## References

[CR1] Andeen NK, Nguyen TQ, Steegh F, Hudkins KL, Najafian B, Alpers CE (2015). The phenotypes of podocytes and parietal epithelial cells may overlap in diabetic nephropathy. Kidney Int.

[CR2] Anders HJ, Fogo AB (2014). Immunopathology of lupus nephritis. Semin Immunopathol.

[CR3] Antonelou M, Evans RDR, Henderson SR, Salama AD (2020) Neutrophils are key mediators in crescentic glomerulonephritis and targets for new therapeutic approaches. Nephrol Dial Transplant. 10.1093/ndt/gfaa206 10.1093/ndt/gfaa206 33057680

[CR4] Appel D, Kershaw DB, Smeets B, Yuan G, Fuss A, Frye B, Elger M, Kriz W, Floege J, Moeller MJ (2009). Recruitment of podocytes from glomerular parietal epithelial cells. J Am Soc Nephrol.

[CR5] Arakawa M, Tokunaga J (1977). A scanning electron microscope study of the human Bowman’s epithelium. Contrib Nephrol.

[CR6] Bariety J, Mandet C, Hill GS, Bruneval P (2006). Parietal podocytes in normal human glomeruli. J Am Soc Nephrol.

[CR7] Benigni A, Morigi M, Rizzo P, Gagliardini E, Rota C, Abbate M, Ghezzi S, Remuzzi A, Remuzzi G (2011). Inhibiting angiotensin-converting enzyme promotes renal repair by limiting progenitor cell proliferation and restoring the glomerular architecture. Am J Pathol.

[CR8] Berger K, Schulte K, Boor P, Kuppe C, van Kuppevelt TH, Floege J, Smeets B, Moeller MJ (2014). The regenerative potential of parietal epithelial cells in adult mice. J Am Soc Nephrol.

[CR9] Bowman W (1842). On the structure and use of the Malphigian bodies of the kidney, with observations on the circulation through that gland. PhilTrans Roy Soc Lond.

[CR10] Burnworth B, Pippin J, Karna P, Akakura S, Krofft R, Zhang G, Hudkins K, Alpers CE, Smith K, Shankland SJ, Gelman IH, Nelson PJ (2012). SSeCKS sequesters cyclin D1 in glomerular parietal epithelial cells and influences proliferative injury in the glomerulus. Lab Invest.

[CR11] Chang AM, Ohse T, Krofft RD, Wu JS, Eddy AA, Pippin JW, Shankland SJ (2012). Albumin-induced apoptosis of glomerular parietal epithelial cells is modulated by extracellular signal-regulated kinase 1/2. Nephrol Dial Transplant.

[CR12] Chen A, Lee K, D'Agati VD, Wei C, Fu J, Guan TJ, He JC, Schlondorff D, Agudo J (2018). Bowman's capsule provides a protective niche for podocytes from cytotoxic CD8+ T cells. J Clin Invest.

[CR13] Couser WG (2012). Basic and translational concepts of immune-mediated glomerular diseases. J Am Soc Nephrol.

[CR14] Cunningham MA, Rondeau E, Chen X, Coughlin SR, Holdsworth SR, Tipping PG (2000). Protease-activated receptor 1 mediates thrombin-dependent, cell-mediated renal inflammation in crescentic glomerulonephritis. J Exp Med.

[CR15] Devi S, Li A, Westhorpe CL, Lo CY, Abeynaike LD, Snelgrove SL, Hall P, Ooi JD, Sobey CG, Kitching AR (2013). Multiphoton imaging reveals a new leukocyte recruitment paradigm in the glomerulus. Nat Med.

[CR16] Dijkman H, Smeets B, van der Laak J, Steenbergen E, Wetzels J (2005 Oct) The parietal epithelial cell is crucially involved in human idiopathic focal segmental glomerulosclerosis. Kidney Int 68(4):1562-72. 10.1111/j.1523-1755.2005.00568.x. PMID: 16164633 10.1111/j.1523-1755.2005.00568.x 16164633

[CR17] Djudjaj S, Lue H, Rong S, Papasotiriou M, Klinkhammer BM, Zok S, Klaener O, Braun GS, Lindenmeyer MT, Cohen CD, Bucala R, Tittel AP, Kurts C, Moeller MJ, Floege J, Ostendorf T, Bernhagen J, Boor P (2016). Macrophage migration inhibitory factor mediates proliferative GN via CD74. J Am Soc Nephrol.

[CR18] Eng DG, Sunseri MW, Kaverina NV, Roeder SS, Pippin JW, Shankland SJ (2015). Glomerular parietal epithelial cells contribute to adult podocyte regeneration in experimental focal segmental glomerulosclerosis. Kidney Int.

[CR19] Eymael J, Sharma S, Loeven MA, Wetzels JF, Mooren F, Florquin S, Deegens JK, Willemsen BK, Sharma V, van Kuppevelt TH, Bakker MA, Ostendorf T, Moeller MJ, Dijkman HB, Smeets B, van der Vlag J (2018). CD44 is required for the pathogenesis of experimental crescentic glomerulonephritis and collapsing focal segmental glomerulosclerosis. Kidney Int.

[CR20] Finsterbusch M, Hall P, Li A, Devi S, Westhorpe CL, Kitching AR, Hickey MJ (2016). Patrolling monocytes promote intravascular neutrophil activation and glomerular injury in the acutely inflamed glomerulus. Proc Natl Acad Sci USA.

[CR21] Froes BP, de Almeida Araújo S, Bambirra EA, Oliveira EA, Silva Simões E, Pinheiro SVB AC (2017). Is CD44 in glomerular parietal epithelial cells a pathological marker of renal function deterioration in primary focal segmental glomerulosclerosis?. Pediatr Nephrol.

[CR22] GBD Chronic Kidney Disease Collaboration (2020). Global, regional, and national burden of chronic kidney disease, 1990–2017: a systematic analysis for the Global Burden of Disease Study 2017. Lancet.

[CR23] Gibson IW, Downie I, Downie TT, Han SW, More IA, Lindop GB (1992). The parietal podocyte: a study of the vascular pole of the human glomerulus. Kidney Int.

[CR24] Gotot J, Piotrowski E, Otte MS, Tittel AP, Linlin G, Yao C, Ziegelbauer K, Panzer U, Garbi N, Kurts C, Thaiss F (2016). Inhibitor of NFκB kinase subunit 2 blockade hinders the initiation but aggravates the progression of crescentic GN. J Am Soc Nephrol.

[CR25] Guettier C, Nochy D, Jacquot C, Mandet C, Camilleri JP, Bariety J (1986). Immunohistochemical demonstration of parietal epithelial cells and macrophages in human proliferative extra-capillary lesions. Virchows Arch A Pathol Anat Histopathol.

[CR26] Henique C, Bollee G, Loyer X, Grahammer F, Dhaun N, Camus M, Vernerey J, Guyonnet L, Gaillard F, Lazareth H, Meyer C, Bensaada I, Legres L, Satoh T, Akira S, Bruneval P, Dimmeler S, Tedgui A, Karras A, Thervet E, Nochy D, Huber TB, Mesnard L, Lenoir O, Tharaux PL (2017). Genetic and pharmacological inhibition of microRNA-92a maintains podocyte cell cycle quiescence and limits crescentic glomerulonephritis. Nat Commun.

[CR27] Heymann F, Meyer-Schwesinger C, Hamilton-Williams EE, Hammerich L, Panzer U, Kaden S, Quaggin SE, Floege J, Gröne HJ, Kurts C (2009) Kidney dendritic cell activation is required for progression of renal disease in a mouse model of glomerular injury. J Clin Invest 119(5):1286–97. 10.1172/JCI38399. Epub 2009 Apr 20. Erratum in: J Clin Invest 119(7):2114 10.1172/JCI38399 PMC2673875 19381017

[CR28] Himmelfarb J, Vanholder R, Mehrotra R, Tonelli M (2020). The current and future landscape of dialysis. Nat Rev Nephrol.

[CR29] Hippen B, Ross LF, Sade RM (2009). Saving lives is more important than abstract moral concerns: financial incentives should be used to increase organ donation. Ann Thorac Surg.

[CR30] Holderied A, Romoli S, Eberhard J, Konrad LA, Devarapu SK, Marschner JA, Müller S, Anders HJ (2015). Glomerular parietal epithelial cell activation induces collagen secretion and thickening of Bowman's capsule in diabetes. Lab Invest.

[CR31] Hooke DH, Gee DC, Atkins RC (1987). Leukocyte analysis using monoclonal antibodies in human glomerulonephritis. Kidney Int.

[CR32] Huang XR, Holdsworth SR, Tipping PG (1994). Evidence for delayed-type hypersensitivity mechanisms in glomerular crescent formation. Kidney Int.

[CR33] Jennette JC, Thomas DB (2001). Crescentic glomerulonephritis. Nephrol Dial Transplant.

[CR34] Kaverina NV, Eng DG, Freedman BS, Kutz JN, Chozinski TJ, Vaughan JC, Miner JH, Pippin JW, Shankland SJ (2019). Dual lineage tracing shows that glomerular parietal epithelial cells can transdifferentiate toward the adult podocyte fate. Kidney Int.

[CR35] Kietzmann L, Guhr SS, Meyer TN, Ni L, Sachs M, Panzer U, Stahl RA, Saleem MA, Kerjaschki D, Gebeshuber CA, Meyer-Schwesinger C (2015). MicroRNA-193a Regulates the transdifferentiation of human parietal epithelial cells toward a podocyte phenotype. J Am Soc Nephrol.

[CR36] Kim S, Kim YH, Choi KH, Jeong HJ (2016). Glomerular epithelial CD44 expression and segmental sclerosis in IgA nephropathy. Clin Exp Nephrol.

[CR37] Kim YH, Goyal M, Kurnit D, Wharram B, Wiggins J, Holzman L, Kershaw D, Wiggins R (2001). Podocyte depletion and glomerulosclerosis have a direct relationship in the PAN-treated rat. Kidney Int.

[CR38] Kitching AR, Hutton HL (2016). The players: cells involved in glomerular disease. Clin J Am Soc Nephrol.

[CR39] Kitching AR, Turner AL, Wilson GR, Semple T, Odobasic D, Timoshanko JR, O'Sullivan KM, Tipping PG, Takeda K, Akira S, Holdsworth SR (2005). IL-12p40 and IL-18 in crescentic glomerulonephritis: IL-12p40 is the key Th1-defining cytokine chain, whereas IL-18 promotes local inflammation and leukocyte recruitment. J Am Soc Nephrol.

[CR40] Kitching AR, Holdsworth SR, Tipping PG (1999). IFN-gamma mediates crescent formation and cell-mediated immune injury in murine glomerulonephritis. J Am Soc Nephrol.

[CR41] Kitching AR, Tipping PG, Holdsworth SR (1999). IL-12 directs severe renal injury, crescent formation and Th1 responses in murine glomerulonephritis. Eur J Immunol.

[CR42] Krebs CF, Schmidt T, Riedel JH, Panzer U (2017). T helper type 17 cells in immune-mediated glomerular disease. Nat Rev Nephrol.

[CR43] Kriz W, Hähnel B, Rösener S, Elger M (1995). Long-term treatment of rats with FGF-2 results in focal segmental glomerulosclerosis. Kidney Int.

[CR44] Kuppe C, Leuchtle K, Wagner A, Kabgani N, Saritas T, Puelles VG, Smeets B, Hakroush S, van der Vlag J, Boor P, Schiffer M, Gröne HJ, Fogo A, Floege J, Moeller MJ (2019). Novel parietal epithelial cell subpopulations contribute to focal segmental glomerulosclerosis and glomerular tip lesions. Kidney Int.

[CR45] Kuppe C, van Roeyen C, Leuchtle K, Kabgani N, Vogt M, Van Zandvoort M, Smeets B, Floege J, Gröne HJ, Moeller MJ (2017). Investigations of glucocorticoid action in GN. J Am Soc Nephrol.

[CR46] Kurts C, Ginhoux F, Panzer U (2020 Jul) Kidney dendritic cells: fundamental biology and functional roles in health and disease. Nat Rev Nephrol 16(7):391-407. 10.1038/s41581-020-0272-y. Epub 2020 May 5. PMID: 32372062 10.1038/s41581-020-0272-y 32372062

[CR47] Kusuyama Y, Nishihara T, Saito K (1981). Nephrotoxic nephritis in nude mice. Clin Exp Immunol.

[CR48] Lasagni L, Lazzeri E, Shankland SJ, Anders HJ, Romagnani P (2013). Podocyte mitosis - a catastrophe. Curr Mol Med.

[CR49] Lazareth H, Lenoir O, Tharaux PL (2020). Parietal epithelial cells role in repair versus scarring after glomerular injury. Curr Opin Nephrol Hypertens.

[CR50] Lazareth H, Henique C, Lenoir O, Puelles VG, Flamant M, Bollée G, Fligny C, Camus M, Guyonnet L, Millien C, Gaillard F, Chipont A, Robin B, Fabrega S, Dhaun N, Camerer E, Kretz O, Grahammer F, Braun F, Huber TB, Nochy D, Mandet C, Bruneval P, Mesnard L, Thervet E, Karras A, Le Naour F, Rubinstein E, Boucheix C, Alexandrou A, Moeller MJ, Bouzigues C, Tharaux PL (2019). The tetraspanin CD9 controls migration and proliferation of parietal epithelial cells and glomerular disease progression. Nat Commun.

[CR51] Luque Y, Cathelin D, Vandermeersch S, Xu X, Sohier J, Placier S, Xu-Dubois YC, Louis K, Hertig A, Bories JC, Vasseur F, Campagne F, Di Santo JP, Vosshenrich C, Rondeau E, Mesnard L (2017). Glomerular common gamma chain confers B- and T-cell-independent protection against glomerulonephritis. Kidney Int.

[CR52] Mazzinghi B, Romagnani P, Lazzeri E (2016). Biologic modulation in renal regeneration. Expert Opin Biol Ther.

[CR53] Moeller MJ, Tharaux PL (2019). Cellular regeneration of podocytes from parietal cells: the debate is still open. Kidney Int.

[CR54] Moeller MJ, Smeets B (2014). Role of parietal epithelial cells in kidney injury: the case of rapidly progressing glomerulonephritis and focal and segmental glomerulosclerosis. Nephron Exp Nephrol.

[CR55] Morigi M, Locatelli M, Rota C, Buelli S, Corna D, Rizzo P, Abbate M, Conti D, Perico L, Longaretti L, Benigni A, Zoja C, Remuzzi G (2016 Jun 27) A previously unrecognized role of C3a in proteinuric progressive nephropathy. Sci Rep. 6:28445. 10.1038/srep28445. PMID: 27345360; PMCID: PMC4921969 10.1038/srep28445 PMC4921969 27345360

[CR56] Neale TJ, Tipping PG, Carson SD, Holdsworth SR (1988). Participation of cell-mediated immunity in deposition of fibrin in glomerulonephritis. Lancet.

[CR57] Nishi H, Furuhashi K, Cullere X, Saggu G, Miller MJ, Chen Y, Rosetti F, Hamilton SL, Yang L, Pittman SP (2017). Neutrophil FcgammaRIIA promotes IgG-mediated glomerular neutrophil capture via Abl/Src kinases. J Clin Invest.

[CR58] Ohse T, Chang AM, Pippin JW, Jarad G, Hudkins KL, Alpers CE, Miner JH, Shankland SJ (2009). A new function for parietal epithelial cells: a second glomerular barrier. Am J Physiol Renal Physiol.

[CR59] Ohse T, Pippin JW, Chang AM, Krofft RD, Miner JH, Vaughan MR, Shankland SJ (2009). The enigmatic parietal epithelial cell is finally getting noticed: a review. Kidney Int.

[CR60] Ohse T, Vaughan MR, Kopp JB, Krofft RD, Marshall CB, Chang AM, Hudkins KL, Alpers CE, Pippin JW, Shankland SJ (2010). De novo expression of podocyte proteins in parietal epithelial cells during experimental glomerular disease. Am J Physiol Renal Physiol.

[CR61] Okamoto T, Sasaki S, Yamazaki T, Sato Y, Ito H, Ariga T (2013). Prevalence of CD44-positive glomerular parietal epithelial cells reflects podocyte injury in adriamycin nephropathy. Nephron Exp Nephrol.

[CR62] Ooi JD, Phoon RK, Holdsworth SR, Kitching AR (2009). IL-23, not IL-12, directs autoimmunity to the good pasture antigen. J Am Soc Nephrol.

[CR63] Pabst R, Sterzel RB (1983). Cell renewal of glomerular cell types in normal rats an autoradiographic analysis. Kidney Int.

[CR64] Paust HJ, Turner JE, Steinmetz OM, Peters A, Heymann F, Hölscher C, Wolf G, Kurts C, Mittrücker HW, Stahl RA, Panzer U (2009). The IL-23/Th17 axis contributes to renal injury in experimental glomerulonephritis. J Am Soc Nephrol.

[CR65] Pichaiwong W, Hudkins KL, Wietecha T, Nguyen TQ, Tachaudomdach C, Li W, Askari B, Kobayashi T, O'Brien KD, Pippin JW, Shankland SJ, Alpers CE (2013). Reversibility of structural and functional damage in a model of advanced diabetic nephropathy. J Am Soc Nephrol.

[CR66] Puelles VG, Cullen-McEwen LA, Taylor GE, Li J, Hughson MD, Kerr PG, Hoy WE, Bertram JF (2016). Human podocyte depletion in association with older age and hypertension. Am J Physiol Renal Physiol.

[CR67] Puelles VG, van der Wolde JW, Wanner N, Scheppach MW, Cullen-McEwen LA, Bork T, Lindenmeyer MT, Gernhold L, Wong MN, Braun F, Cohen CD, Kett MM, Kuppe C, Kramann R, Saritas T, van Roeyen CR, Moeller MJ, Tribolet L, Rebello R, Sun YB, Li J, Müller-Newen G, Hughson MD, Hoy WE, Person F, Wiech T, Ricardo SD, Kerr PG, Denton KM, Furic L, Huber TB, Nikolic-Paterson DJ, Bertram JF (2019a) mTOR-mediated podocyte hypertrophy regulates glomerular integrity in mice and humans. JCI Insight 4(18):e99271. 10.1172/jci.insight.99271 PMC6795295 31534053

[CR68] Puelles VG, Moeller MJ (2019b) Postnatal podocyte gain: Is the jury still out? Semin Cell Dev Biol 91:147–152 10.1016/j.semcdb.2018.07.007 31178004

[CR69] Puelles VG, Fleck D, Ortz L, Papadouri S, Strieder T, Böhner AMC, van der Wolde JW, Vogt M, Saritas T, Kuppe C, Fuss A, Menzel S, Klinkhammer BM, Müller-Newen G, Heymann F, Decker L, Braun F, Kretz O, Huber TB, Susaki EA, Ueda HR, Boor P, Floege J, Kramann R, Kurts C, Bertram JF, Spehr M, Nikolic-Paterson DJ, Moeller MJ (2019). Novel 3D analysis using optical tissue clearing documents the evolution of murine rapidly progressive glomerulonephritis. Kidney Int.

[CR70] Roeder SS, Barnes TJ, Lee JS, Kato I, Eng DG, Kaverina NV, Sunseri MW, Daniel C, Amann K, Pippin JW, Shankland SJ (2017). Activated ERK1/2 increases CD44 in glomerular parietal epithelial cells leading to matrix expansion. Kidney Int.

[CR71] Romoli S, Angelotti ML, Antonelli G, Kumar Vr S, Mulay SR, Desai J, Anguiano Gomez L, Thomasova D, Eulberg D, Klussmann S, Melica ME, Conte C, Lombardi D, Lasagni L, Anders HJ, Romagnani P (2018). CXCL12 blockade preferentially regenerates lost podocytes in cortical nephrons by targeting an intrinsic podocyte-progenitor feedback mechanism. Kidney Int.

[CR72] Ronconi E, Sagrinati C, Angelotti ML, Lazzeri E, Mazzinghi B, Ballerini L, Parente E, Becherucci F, Gacci M, Carini M, Maggi E, Serio M, Vannelli GB, Lasagni L, Romagnani S, Romagnani P (2009). Regeneration of glomerular podocytes by human renal progenitors. J Am Soc Nephrol.

[CR73] Ryu M, Migliorini A, Miosge N, Gross O, Shankland S, Brinkkoetter PT, Hagmann H, Romagnani P, Liapis H, Anders HJ (2012). Plasma leakage through glomerular basement membrane ruptures triggers the proliferation of parietal epithelial cells and crescent formation in non-inflammatory glomerular injury. J Pathol.

[CR74] Sagrinati C, Netti GS, Mazzinghi B, Lazzeri E, Liotta F, Frosali F, Ronconi E, Meini C, Gacci M, Squecco R, Carini M, Gesualdo L, Francini F, Maggi E, Annunziato F, Lasagni L, Serio M, Romagnani S, Romagnani P (2006). Isolation and characterization of multipotent progenitor cells from the Bowman's capsule of adult human kidneys. J Am Soc Nephrol.

[CR75] Sato T, Oite T, Nagase M, Shimizu F (1991). Nephrotoxic serum nephritis in nude rats: the roles of host immune reactions. Clin Exp Immunol.

[CR76] Schulte K, Berger K, Boor P, Jirak P, Gelman IH, Arkill KP, Neal CR, Kriz W, Floege J, Smeets B, Moeller MJ (2014). Origin of parietal podocytes in atubular glomeruli mapped by lineage tracing. J Am Soc Nephrol.

[CR77] Shankland SJ, Smeets B, Pippin JW, Moeller MJ (2014). The emergence of the glomerular parietal epithelial cell. Nat Rev Nephrol.

[CR78] Shankland SJ, Freedman BS, Pippin JW (2017). Can podocytes be regenerated in adults?. Curr Opin Nephrol Hypertens.

[CR79] Sicking EM, Fuss A, Uhlig S, Jirak P, Dijkman H, Wetzels J, Engel DR, Urzynicok T, Heidenreich S, Kriz W, Kurts C, Ostendorf T, Floege J, Smeets B, Moeller MJ (2012). Subtotal ablation of parietal epithelial cells induces crescent formation. J Am Soc Nephrol.

[CR80] Smeets B, Uhlig S, Fuss A, Mooren F, Wetzels JF, Floege J, Moeller MJ (2009). Tracing the origin of glomerular extracapillary lesions from parietal epithelial cells. J Am Soc Nephrol.

[CR81] Smeets B, Angelotti ML, Rizzo P, Dijkman H, Lazzeri E, Mooren F, Ballerini L, Parente E, Sagrinati C, Mazzinghi B, Ronconi E, Becherucci F, Benigni A, Steenbergen E, Lasagni L, Remuzzi G, Wetzels J, Romagnani P (2009). Renal progenitor cells contribute to hyperplastic lesions of podocytopathies and crescentic glomerulonephritis. J Am Soc Nephrol.

[CR82] Smeets B, Kuppe C, Sicking EM, Fuss A, Jirak P, van Kuppevelt TH, Endlich K, Wetzels JF, Gröne HJ, Floege J, Moeller MJ (2011). Parietal epithelial cells participate in the formation of sclerotic lesions in focal segmental glomerulosclerosis. J Am Soc Nephrol.

[CR83] Smeets B, Stucker F, Wetzels J, Brocheriou I, Ronco P, Gröne HJ, D'Agati V, Fogo AB, van Kuppevelt TH, Fischer HP, Boor P, Floege J, Ostendorf T, Moeller MJ (2014). Detection of activated parietal epithelial cells on the glomerular tuft distinguishes early focal segmental glomerulosclerosis from minimal change disease. Am J Pathol.

[CR84] Suh KS, Kim BK, Kim KH (1999). Crescentic glomerulonephritis: a clinicopathologic analysis of 17 cases with emphasis on glomerular and interstitial neutrophil infiltration. J Kor Med Sci.

[CR85] Tang PM, Nikolic-Paterson DJ, Lan HY (2019). Macrophages: versatile players in renal inflammation and fibrosis. Nat Rev Nephrol.

[CR86] Taugner R, Boll U, Zahn P, Forssmann WG (1976). Cell junctions in the epithelium of Bowman’s capsule. Cell Tissue Res.

[CR87] Tipping PG, Holdsworth SR (2006). T cells in crescentic glomerulonephritis. J Am Soc Nephrol.

[CR88] van den Berg JG, Weening JJ (2004). Role of the immune system in the pathogenesis of idiopathic nephrotic syndrome. Clin Sci (lond).

[CR89] Wanner N, Hartleben B, Herbach N, Goedel M, Stickel N, Zeiser R, Walz G, Moeller MJ, Grahammer F, Huber TB (2014). Unraveling the role of podocyte turnover in glomerular aging and injury. J Am Soc Nephrol.

[CR90] Wharram BL, Goyal M, Wiggins JE, Sanden SK, Hussain S, Filipiak WE, Saunders TL, Dysko RC, Kohno K, Holzman LB, Wiggins RC (2005). Podocyte depletion causes glomerulosclerosis: diphtheria toxin-induced podocyte depletion in rats expressing human diphtheria toxin receptor transgene. J Am Soc Nephrol.

[CR91] Wiggins RC (2007). The spectrum of podocytopathies: a unifying view of glomerular diseases. Kidney Int.

[CR92] Zimmermann M, Klaus M, Wong MN, Thebille AK, Gernhold L, Kuppe C, Halder M, Kranz J, Wanner N, Braun F, Wulf S, Wiech T, Panzer U, Krebs CF, Hoxha E, Kramann R, Huber TB, Bonn S, Puelles VG (2021 Apr 8) Deep learning-based molecular morphometrics for kidney biopsies. JCI Insight 6(7):e144779. 10.1172/jci.insight.144779. PMID: 33705360; PMCID: PMC8119189 10.1172/jci.insight.144779 PMC8119189 33705360

